# Discordance between radiological and pathological response to neoadjuvant immunotherapy in mismatch repair-deficient/microsatellite instability-high colorectal cancer: a meta-analysis

**DOI:** 10.3389/fimmu.2026.1680500

**Published:** 2026-02-09

**Authors:** Yilin Xie, Leen Liao, Peirong Ding, Wu Jiang

**Affiliations:** 1 Department of Colorectal Surgery, State Key Laboratory of Oncology in South China, Guangdong Provincial Clinical Research Center for Cancer, Sun Yat-sen University Cancer Center, Guangzhou, China; 2 Sun Yat-sen University Cancer Center, Guangzhou, China; 3 State Key Laboratory of Oncology in South China, Guangzhou, China; 4 Collaborative Innovation Center for Cancer Medicine, Guangzhou, China

**Keywords:** dMMR/MSI-H colorectal cancer, meta-analysis, microsatellite instability-high, neoadjuvant immunotherapy, radiological assessment

## Abstract

**Background:**

Mismatch repair deficiency (dMMR) and microsatellite instability (MSI-H) cancers exhibit high immunogenicity and are highly responsive to immune checkpoint inhibitors. In patients with locally advanced dMMR/MSI-H colorectal cancer (CRC), neoadjuvant immunotherapy (NIT) has demonstrated unprecedented pathological complete response (pCR) rates, suggesting nonoperative management strategies may be possible. There remains a discrepancy between radiological assessment and pathological responses to NIT in CRC.

**Methods:**

We conducted a systematic review and meta-analysis of studies published between February 2015 and February 2025 to determine if radiological and pathological assessments following neoadjuvant immune checkpoint inhibitor therapy (NIT) were consistent in patients with non-metastatic dMMR/MSI-H CRC. Using PubMed, Embase, and Web of Science, the literature was retrieved, with inclusion criteria focusing on studies that reported both imaging data and pathological results. A random-effects model was used to calculate pooled odds ratios (ORs) and 95% confidence intervals (CIs). Subgroup analyses were conducted based on tumor location (colon versus rectum) and type of response (cCR versus pCR).

**Results:**

In 12 studies, 396 patients were included. A total rate of 59.6% discordance was found between radiological and pathological responses. Compared to rectal cancer patients (34.9%), colon cancer patients exhibited a significantly higher rate of discordance (64.2%). A total of 238 patients with confirmed pCR were incorrectly diagnosed as having residual disease on radiological assessment (OR = 61.41; 95% CI: 10.05–375.27;P < 0.00001). A high level of heterogeneity was observed across studies (I2 = 85%), but no publication bias was observed.

**Conclusion:**

In dMMR/MSI-H CRC, radiologic assessment alone cannot reliably assess the efficacy of NIT, particularly in colon cancer. It should be integrated with additional modalities—such as endoscopic evaluation and biomarker analysis—to ensure an accurate appraisal of treatment efficacy.

## 1 Introduction

Mismatch repair-deficient (dMMR) or microsatellite instability-high (MSI-H) colorectal cancer (CRC) represents a distinct CRC subtype characterized by high immunogenicity and a high level of lymphocyte infiltration, which leads to increased sensitivity to immune checkpoint inhibitors. As a result, Immune checkpoint inhibitor has been recommended as the first-line therapy in patients with metastatic dMMR/MSI-H CRC (1, 2). Recently, efforts to evaluate immune checkpoint inhibitors for locally advanced disease have been pursued. In the NICHE-3 and PICC trials, neoadjuvant immunotherapy (NIT) showed strikingly high response rates, with the pathological complete response (pCR) rate ranging from 68% to 76% in locally advanced dMMR/MSI-H CRC (3–5).

Given the high pCR rates, several studies have explored the possibility of immune checkpoint inhibitors as an alternative to radical surgery for patients with locally advanced dMMR/MSI-H CRC. A phase 2 trial conducted by Cercek et.al (6) found that 49 patients with localized dMMR rectal cancer all achieved clinical complete response (cCR) and were managed with a watch-and-wait (W&W) strategy. Over a median follow-up period of 26.3 months, none of these patients experienced tumor recurrence. This result was echoed by our retrospectively research, in which the 3-year disease-free and overall survivals were both 100% (7). Similar results were also observed in dMMR/MSI-H colon cancer (6). The NEOCAP trail (8) reported that 16 patients with colon cancer and four with multiple primary CRC achieved cCR. and were managed with a W&W strategy. With median follow-up time of 11.2 months since reaching cCR, no tumor regrowth was observed in any of these patients.

In the era of immunotherapy, the treatment of CRC prioritized organ function preservation, leading to higher demands on the accuracy of imaging assessment, especially for those achieved complete response. However, tumor responses to NIT are more prone to be underestimated by radiological assessment. Specifically, patients with pCR were often evaluated as having residual diseases on CT scans. In the PICC trail (9), 80% (24/30) of patients with partial response (PR) were found to achieve pCR after tumor resection. Similarly, in the NICHE-2 study (10), only 2 out of 75 patients with pCR had a radiological complete response, highlighting the poor correlation between radiological assessment and pathological results in colon cancer. However, this phenomenon in rectal cancer was quite different. Cercek et al. reported that rectal cancer patients who achieved cCR with dostarlimab treatment also demonstrated complete response in radiological assessments (11).

There remains a discrepancy between radiological and responses to NIT in CRC, because individual trials lacked sufficient events to provide conclusive insights. This study aims to comparatively analyze the correlation between radiological evaluations and pathological responses in such patients who achieved complete remission after NIT.

## 2 Methods

### 2.1 Literature search

A systematic search was conducted in PubMed, Embase, and Web of Science for studies published from February 22nd, 2015 to February 8, 2025, to identify all published studies examining the efficacy of NIT on CRC. Detailed information on the search strategy is included in the **Supplementary Appendix eMethod**. The research protocol was prospectively registered in the International Prospective Register of Systematic Reviews (PROSPERO) registry (CRD420251090586).

### 2.2 Screening process and eligibility criteria

Two independent reviewers (YLX and LEL) conducted a blinded screening of titles/abstracts identified in the initial search based on predefined eligibility criteria. We included randomized controlled trials (RCTs), prospective/retrospective cohort studies, and single-arm trials investigating NIT with subsequent radiological response assessment in non-metastatic CRC with dMMR or MSI-H. The primary exclusion criteria were (1) reviews, case reports, animal research, or conference abstracts lacking full-text data, and (2) studies with fewer than 15 participants. Discrepancies were adjudicated by a third investigator (WJ). Articles meeting the inclusion criteria during the title/abstract screening phase were retrieved for full-text review, and the same two investigators reviewed the full-text publications to determine eligibility. Relevant systematic reviews were identified and assessed during the full-text assessment phase of the current analysis to validate the search strategy and identify potentially missed publications. Discrepancies were adjudicated by a third investigator (WJ). Given the possible overlap between cohort studies and randomized controlled studies, a thorough study-mapping exercise was performed on full-text and **Supplementary Materials** to identify publications reporting the same study using unique identifiers, including registration number, authorship, and sample size. This approach ensured that reported outcomes from different sources were collected from distinct patient populations, avoiding outcome duplication in the final dataset.

### 2.3 Data extraction and quality assessment

The extracted data included the study design, patient characteristics, primary outcomes, pathological complete response (pCR), clinical complete response (cCR), and secondary outcomes, such as partial response (PR), objective response rate (ORR). Discrepancies between the findings of the two investigators were discussed to resolve. Clinical data are summarized in **Table 1** and **Table 2**.

**Table 1:** Characteristics of the included articles.

Study	Case	Age	Sex (female)	Country	MMR/MSI status	Tumor stage	Tumor location	N stage	Lynch syndrome	ECOG (0-1)	Median follow-up(month)	Radiological assessment	PCR	CCR	PR	R0
Cao 2023 (5)	36	NR	NR	China	36	T1:0 T2:0 T3:6 T4:30	Left colon:7 Right colon:17 transverse: rectum:4	N0:4 N+:32	NR	NR	NR	CT	28	0	NR	36
Chalabi 2024	115	60	67	Netherlands	115	T1:0 T2:17 T3:24 T4:74	Left colon:20 Right colon:78 transverse: 17 rectum:0	N0:38 N+:77	37	NR	26	CT	75	2	4	115
Fox 2023 (12)	38	61	11	USA	38	T1:2 T2:1 T3:17 T4:13	Left colon:7 Right colon:12 transverse:0 rectum:17	N0:5 N+:29	15	NR	28.2	CT 、MRI	11	16	20	18
Li T 2024	24	49	5	China	21	T1:0 T2:1 T3:17 T4:6	Left colon:5 Right colon:8 transverse:3 rectum:5	N0:5 N+:19	12	NR	9.7	CT 、MRI	10	3	4	20
Li Y 2023 (13)	36	10	52	China	30	T1:0 T2:1 T3:21 T4:14	Left colon:NR Right colon:NR transverse:NR rectum:7	N0:5 N+:31	16	32	9.4	CT、MRI、PET-CT	13	7	6	29
Li L 2024	24	50	6	China	20	T1:0 T2:2 T3:6 T4:14	Left colon:3 Right colon:8 transverse:3 rectum:3	N0:1 N+:21	10	NR	16	CT、MRI、PET-CT	14	2	4	20
Yang 2023 (14)	20	55	7	China	20	T1:0 T2:2 T3:7 T4:11	Left colon:0 Right colon:0 transverse:0 rectum:20	N0:6 N+:14	2	20	24.3	MRI、TRUS、CT	11	7	9	13
Zhang 2022 (15)	32	44	15	China	32	T1:0 T2:0 T3:6 T4:26	Left colon:7 Right colon:2 transverse:0 rectum:8	N0:4 N+:28	9	32	14	CT、MRI、TRUS	22	3	29	29
Deng 2024 (16)	20	56	9	China	20	T1:0 T2:0 T3:9 T4:7	Left colon:NR Right colon:NR transverse:NR rectum:5	N0:1 N+:17	5	NR	NR	CT 、MRI	15	2	18	NR
Wang 2022 (17)	25	48	9	China	19	T1:0 T2:3 T3:10 T4:6	Left colon:0 Right colon:0 transverse:0 rectum:19	N0:5 N+:14	12	NR	20.9	CT 、MRI	5	20	20	5
Chen 2023	17	50	6	China	17	T1:0 T2:2 T3:10 T4:5	Left colon:0 Right colon:0 transverse:0 rectum:17	N0:3 N+:14	6	NR	17.2	CT 、MRI	6	9	NR	6
Xiao 2023 (18)	73	48	29	China	73	T1:0 T2:3 T3:22 T4:29	Left colon:16 Right colon:32 transverse:14 rectum:18	N0:7 N+:66	27	73	13.4	NR	28	17	45	49

PCR, pathological complete response; CCR, complete clinical response; PR, partial response; NR, no record; R0,negative surgical margin

**Table 2:** Clinical stage, T stage, N stage of the included articles.

Study	Clinical stage				Clinical T stage		Clinical N stage	
	I	II	III	IV	T1- T2	T3- T4	N0	N+
Cao et al.	NR	NR	NR	NR	0	36	4	32
Chalab et al.	NR	NR	NR	NR	98	17	38	77
Chen et al.	0	0	3	14	0	17	3	14
Deng et al.	0	1	16	3	0	16	1	17
Fox et al.	2	1	32	3	3	30	5	29
Li L et al.	0	1	14	3	2	20	1	21
Li T et al.	0	0	17	7	1	23	5	19
Li Y et al.	0	27	NR	9	1	35	5	31
Xiao et al.	NR	NR	NR	NR	3	51	7	66
Wang et al.	1	4	14	0	3	16	5	14
Yang et al.	1	4	14	0	2	18	6	14
Zhang et al.	0	4	28	0	0	32	4	28

Based on the Methodological Index for Nonrandomized Studies (MINORS) scale (19), two investigators independently searched the relevant literature and evaluated the quality of the selected clinical data. In total, eight domains were analyzed: research purpose, patients’ enrollment, data collection, outcomes, assessment bias, follow-up time, loss rate, and the estimation of sample size (**Supplementary Figure S1**). Sensitivity analyses were performed on the outcome to assess the impact of various variables on sensitivity and trends. Statistical significance was set at a P value of.05.

### 2.4 Consistency definition

The consistency of radiological and pathological results was defined based on the following: (1) Patients achieve radiological complete response and pCR confirmed by postoperative pathological examinations; (2) Preoperative imaging identified residual disease, and postoperative pathological examination detected residual tumor; (3) Given the application of W&W strategies after NIT, if patient achieved cCR and has not experienced recurrence for more than 6 months, these patients were considered as having achieved pCR. pCR was defined as no residual invasive disease in either primary tumor or lymph nodes after NIT. The evaluation of cCR is primarily defined by the researchers in respective studies.

### 2.5 Statistical analysis

In our study, we performed a meta-analysis using STATA (version 17; StataCorp LLC, College Station, TX) and Review Manager (RevMan) software version 5.4 (Cochrane Collaboration). We calculated the relative risk (OR) for binary variables, accompanied by their respective 95% CIs. We assessed the presence of between-study heterogeneity using the Cochran Q and I^2^ tests, considering a significance level of 2-sided p < 0.05 for the Q statistic and I^2^ values above 40% as indicative of significant heterogeneity. And, random-effect model was employed if there was heterogeneity between studies (p < 0.05 or I^2^ > 50%). Publication bias was assessed through funnel plots and Egger tests. Moreover, sensitivity analysis was performed using the leave-one-out method.

## 3 Results

### 3.1 Study selection and evaluation

**Figure 1:**
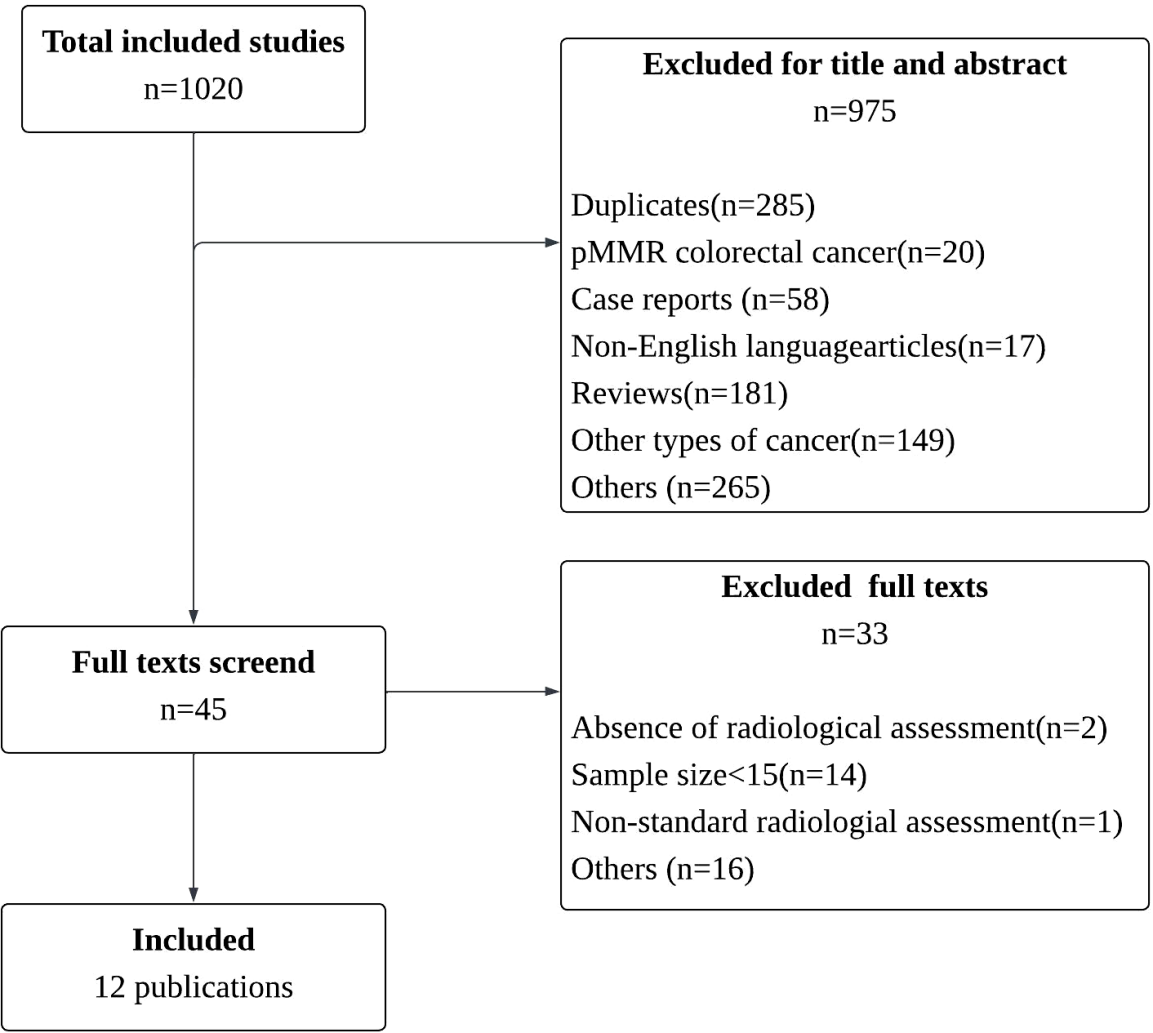
Study selection flowchart.

Searches of medical databases, including Web of Science, Embase, and PubMed, resulted in the identification of 1020 relevant records. After a comprehensive screening of titles and abstracts and removed duplicate entries, we refined the list to 735 studies. During the screening of titles and abstracts, 690 records were excluded, leaving 45 records that were assessed in full text to confirm their eligibility (**Figure 1**). Finally, 12 articles qualified for the meta-analysis were included (5, 12–18, 20–23). A comprehensive summary of the baseline characteristics of the 12 included studies is presented in **Table 1**.

### 3.2 Study characteristics

A total of ten retrospective observational studies and two clinical trials were selected from publications published between July 2022 and March 2025. The sample size ranged from 17 to 111 cases, with a total of 396 patients included, of whom 169 had colon cancer and 227 had rectal cancer. Among the included studies, mostly PD-1 inhibitor monotherapy (e.g., pembrolizumab) or combination therapy with CTLA-4 inhibitors (e.g., ipilimumab) or chemotherapy (e.g., FOLFOX) was used as a neoadjuvant treatment regimen, with some rectal patients receiving concurrent radiotherapy as well. In different studies, treatment schedules varied, with some protocols following fixed cycles (e.g., every three weeks for two to four cycles), and others adjusting treatment schedules in response to efficacy assessment. W&W strategies were used for patients who achieved cCR. Detailed information regarding the selected studies is provided in the **Supplementary Appendix eTable 1** and **eTable 2**.

A low risk of publication bias was found by the funnel plots among included studies. It has been reported that, however, the Egger’s test indicates no significant bias among the studies (**Supplementary Appendix eFigures 2**; **eTable 3**). In the sensitivity analysis, with leave-one-out method, the results of the remaining studies were not statistically significant. But it found that two studies employed the unique enrollment and exclusion criteria during the therapy period. Chen et.al (21) employed stringent screening criteria, excluding patients who exhibited tumor shrinkage of less than 20%, tumor enlargement, or metastasis following the first two cycles of initial therapy. Moreover, both Chen et.al (21) and Wang et al. (17) exclusively enrolled patients with rectal cancer. After excluding these two studies and re-conducting the sensitivity analysis, the results demonstrated robustness and were not easily influenced by individual studies. (**Supplementary Appendix eFigures 3**).

### 3.3 The result of the consistency evaluation

**Figure 2:**
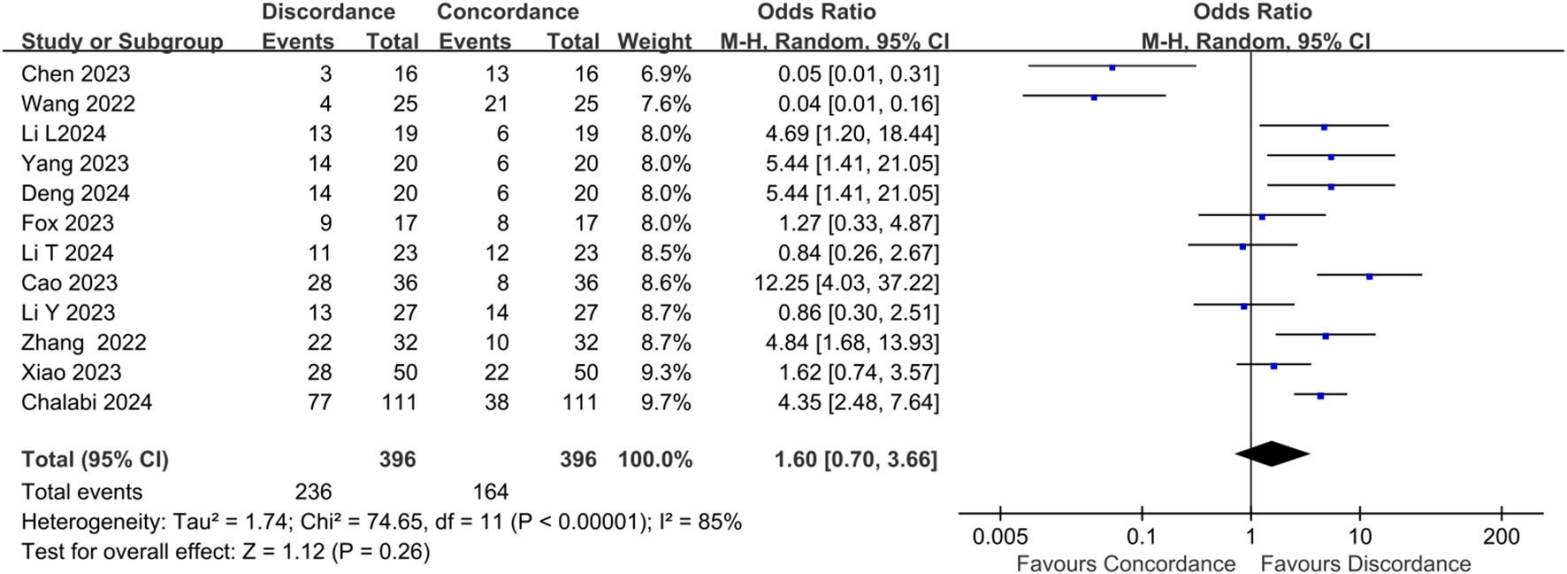
Discordance of radiological and pathological assessment in CRC.

All 12 studies shared their findings on radiological and pathological results. There was a high heterogeneity among these studies, with I²= 85% and P <0.00001. Based on the random-effects model, 236 out of 396 patients (59.6%) had inconsistent results between radiological and pathological results. (OR: 1.60, 95%CI: 0.70-3.66, P = 0.26, Chi = 74.65, **Figure 2**). Notably, 47 patients whose radiological evaluation indicated clinical complete response were no recurrence within 6 months or were confirmed as pCR (OR: 0.02, 95% CI: 0.01–0.09, P <0.00001, Chi² = 4.36, **eFigure S4**). Only 2 cases with MRI-indicated cCR had residual tumors in postoperative pathology. Conversely, 219 of the 238 patients who achieved pCR showed results inconsistent with their radiological assessment(OR: 61.41, 95% CI: 10.05–375.27, P <0.00001, Chi² = 63.99, **eFigure S5**).

### 3.4 Subgroup analyses of colon cancer and rectal cancer

**Figure 3:**
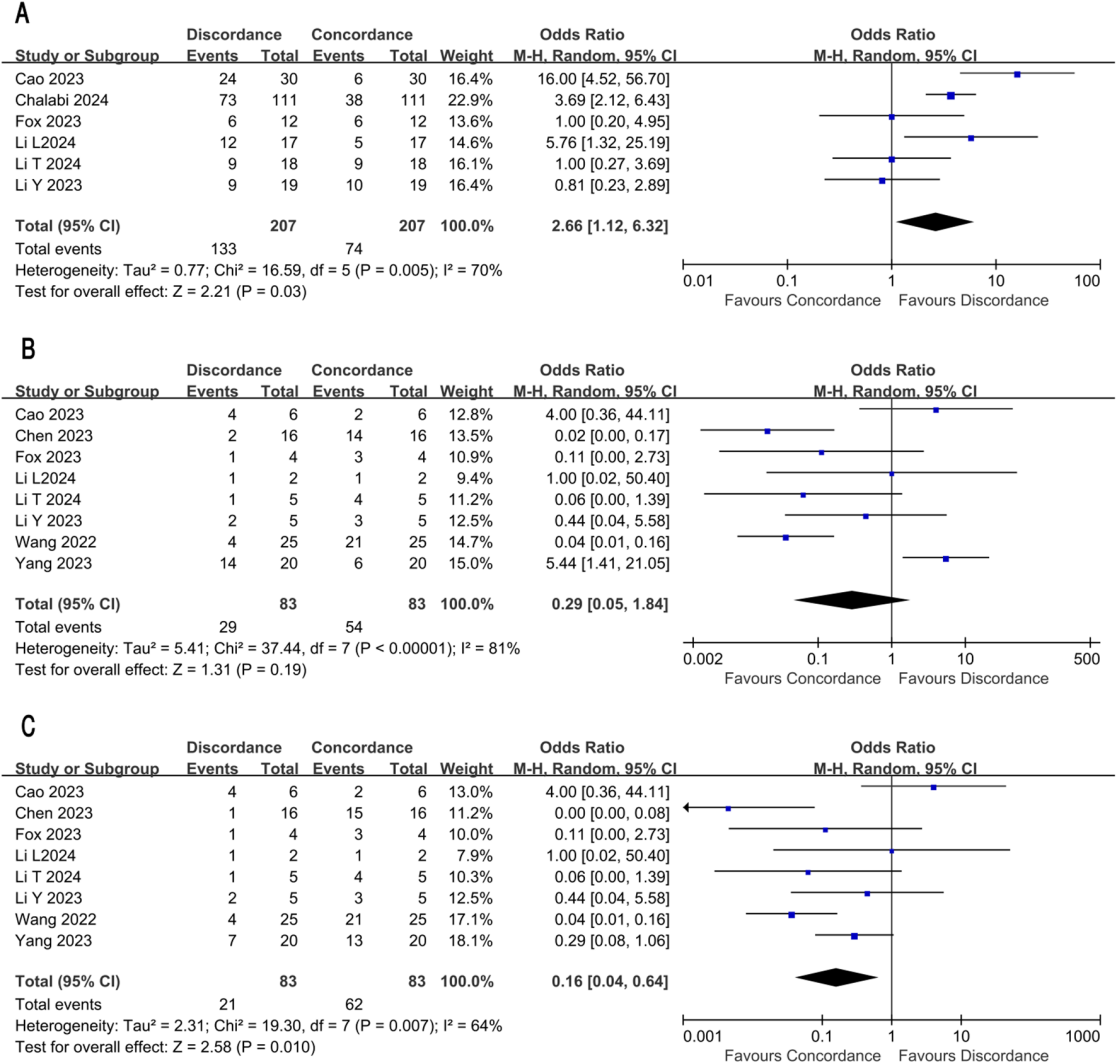
Discordance of radiological and pathological assessment in subgroup **(A)** Discordance in colon cancer **(B)** Discordance in rectal cancer **(C)** Discordance in rectal cancer after re-classification.

For colon cancer, results from 6 studies involving 207 patients were included. There was a high heterogeneity among these studies, with I²= 70% and *P* = 0.005. Based on the random-effects model, 133/207(64.3%) patients had inconsistent results (OR:2.66, 95% CI: 1.12-6.32, P = 0.03, Chi =16.59, **Figure 3A**). Among 136 colon cancer patients who achieved pCR, only 4 cases in the subgroup were reported as cCR.

For rectal cancer, results from 8 studies involving 83 patients were included. There was a high heterogeneity among these studies, with I²= 81% and *P* < 0.00001. Based on the random-effects model, 29/83(34.9%) patients had inconsistent results (OR:0.29, 95% CI: 0.05-1.84, P = 0.19, Chi = 37.44, **Figure 3B**). Among 25 rectal cancer patients who achieved pCR, only 4 cases were reported as cCR, while 39 of 41 rectal cancer patients who achieved cCR had pCR or no recurrence within 6 months.

After classified near-cCR as equivalent to cCR, we re-ran the meta-analysis on the rectal cancer subtype (**Figure 3C**). Under this definition, discordant cases fell from 29/83 (34.9%) to 21/83 (25.3%) (OR: 0.16, 95% CI: 0.04–0.64, P = 0.010, Chi² = 19.30, **Figure 3C**), with moderate residual heterogeneity (I² = 64%, P = 0.007).

## 4 Discussion

The W&W strategy, as a non-surgical treatment, holds the potential of organ preservation and quality of life improvement. Given that dMMR/MSI-H tumor patients exhibit significantly higher pCR rates after NIT, this subtype is most likely to benefit from a W&W strategy (24–26). Therefore, accurately distinguishing patients who achieve pCR from those with residual lesions is critical. Only through precise assessment of treatment response can these patients be safely diverted from surgery. This meta-analysis involving 396 patients evaluated the efficacy of NIT in dMMR/MSI-H CRC. Radiological assessment results showed significant discrepancies with pathological findings, with an overall inconsistency rate of 59.6%. This phenomenon was more pronounced in colon cancer patients, where the inconsistency rate reached 64.2%. Thus, the study indicates that current imaging modalities are insufficient to meet the clinical need.

After rigorous screening and quality assessment, the results of the study were reliable, despite a high degree of heterogeneity among the studies (I^2^ = 85%). The reliability of this study was further confirmed by sensitivity analysis and funnel plot assessment, which showed no significant publication bias (Egger’s test P>0.05). Among 238 patients who achieved pathological complete response (pCR), only 19 were classified as complete response (CR) on radiological assessment. And among 47 patients who achieved clinical complete response (cCR), only 2 cases had residual tumors in postoperative pathology. It indicates that radiological assessment underestimates the efficacy of NIT in dMMR/MSI-H CRC. This phenomenon may be attributed to the changes in the immune microenvironment. Yumo et al. (27) suggests that immunotherapy causes more fibrosis and more dense immune cell infiltration in the tumor microenvironment, leading to pseudoprogression and pseudoresidue. Therefore, residual disease on radiological scans does not indicate viable tumor cells after NIT.

It should be noted that subgroup analysis revealed a high inconsistency rate of 64.3% in the colon cancer group, while the rectal cancer group showed only 34.5%. Moreover, there is a substantial proportion of rectal cancer showed the near-cCR on radiological assessment. After considering these patients as equivalent to cCR, results demonstrated a robust concordance between radiological and pathological assessments in rectal cancer (P = 0.010), after reclassification. This may be caused by following reasons: First, the embryonic origin and developmental rate of the colon and rectum differ, leading to significant variations in immune cell characteristics. Colon tissue exhibits higher immune cell infiltration, whereas immune cells in the rectum are primarily confined to the mucosal layer (28). As a result, colon cancer patients are more likely to have residual masses after NIT. Second, rectal cancer is typically evaluated using MRI, which provides more detailed imaging information than CT. Additionally, digital rectal examination allows clinicians to assess treatment outcomes more intuitively.

Recently, other approaches have been explored for evaluating the efficacy of NIT in colon cancer. As demonstrated in the NEST-1 study (29, 30) unlike traditional chemotherapy, the response pattern to immunotherapy is “serum to mucosa,” where immune cells penetrate the cancer cells deep within the body. In the Ludford et al. study (31) of 27 MSI-H CRC patients, the luminal endoscopic response was as high as 89%, which illustrated that endoscopic assessment may provide additional effective evaluation of therapy response. In Cercek et al. study (6), endoscopy combined with MRI, PET-CT, endoscopic ultrasound (EUS), and other imaging modalities were used to jointly assess the NIT efficacy. The results showed that among all rectal cancer patients who achieved cCR (n = 49), only one experienced recurrence; the remaining patients did not relapse during the follow-up period, including 37 who had been followed over 12 months. In addition, dynamic changes in quantitative ctDNA levels showed high concordance with radiological assessments: in all patients with respond, ctDNA levels fell by 83–100%, aligning closely with radiological results (32). During a period of radiographic pseudoprogression, sustained ctDNA declines continued to accurately reflect true treatment benefit (32). Moreover, the BESPOKE CRC study highlighted ctDNA’s prognostic power in predicting recurrence risk for patients with CRC. Circulating tumor DNA positivity strongly correlated with inferior DFS, which provides a new research direction for the evaluation of immunotherapy. To sum up, the optimal approach to efficacy evaluation has significant clinical implications for the achievement of organ preservation during NIT treatment of colon cancer. Despite its limitations, radiology, as a foundational assessment, can be combined with other methods to jointly evaluate treatment efficacy, thereby improving predictive accuracy.

To sum up, selecting the optimal approach for evaluating efficacy holds significant clinical importance for achieving organ preservation during NIT treatment of CRC. Current definitions of cCR vary significantly based on tumor location. For rectal cancer, cCR is rigorously defined by a multimodal assessment comprising digital rectal examination (DRE), endoscopic evaluation, and MRI (6). In contrast, the definition of cCR for colon cancer has not yet been standardized. However, it is intuitive that an accurate assessment for colon cancer should emulate the rigor of rectal protocols by integrating endoscopic assessment with cross-sectional imaging, thereby preventing the underestimation of residual disease. Despite its limitations, radiology, as a foundational assessment, can be combined with these methods to jointly evaluate treatment efficacy, which in turn enhances the accuracy of predicting treatment outcomes.

This study has several limitations: Firstly, the majority of the included studies had small sample sizes, which may introduce random error and selection bias. The robustness of our conclusions should be confirmed in larger prospective cohorts. Second, the proportion of colon versus rectal cancer patients varied considerably across studies, which may affect the comparability of efficacy assessments. Third, the various radiological diagnostic equipment used in the included studies may lead to biases in radiological results, thereby affecting the evaluation of the efficacy of neoadjuvant immunotherapy. Fourth, most of the included studies originated from Asian centers. This geographic concentration suggests that our findings may not be fully generalizable to global populations due to potential differences in clinical practice patterns and patient genetic backgrounds. In addition, to ensure the reliability of concordance assessments, non-surgical patients whose follow-up did not reach 6 months were not included in the analysis, which may further introduce selection bias.

However, we systematically identified all available studies that included both radiological and pathological assessments, and by conducting rigorous quality assessment and publication bias analyses, we ensured the stability and reliability of our meta-analytic results.

### 4.1 Novelty of the study

Several studies have shown differences between radiology results and pathology results after neoadjuvant immunotherapy in CRC, but there has not been a systematic review. This study uses meta-analysis to see if imaging response matches pathological response in dMMR CRC after neoadjuvant immunotherapy. It provides a systemized and objective evaluation of the difference in assessment between imaging and pathology to better measure these differences. Additionally, subgroup analyses were performed to examine how anatomical location and post-treatment surgery on the tumor may affect the outcome. It revealed an overall discordance rate of 59.6% between radiological and pathological assessments. Colon cancers exhibited a higher discordance rate (64.2%), whereas rectal cancers showed greater concordance, with a discordance rate of only 34.9%. By looking at the results, future studies may get more ideas for developing new imaging biomarkers and better therapeutic strategies.

### 4.2 Conclusion

In dMMR/MSI-H CRC, radiologic assessment alone cannot reliably assess the efficacy of NIT, particularly in colon cancer. For rectal cancer, radiologic assessment exhibited a tendency toward concordance, despite not achieving statistical significance (p =0.19). To select optimal post-treatment regimens and pursue non-operative management, radiologic assessment should be integrated with additional modalities—such as endoscopic evaluation and biomarker analysis—to ensure an accurate appraisal of treatment efficacy.
