# Supplementary material for: Discordance between radiological and pathological response to neoadjuvant immunotherapy in mismatch repair-deficient/microsatellite instability-high colorectal cancer: a meta-analysis
Source: Front Immunol. 2026 Feb 9;17:1680500. doi: 10.3389/fimmu.2026.1680500 (PMC12926347; doi:10.3389/fimmu.2026.1680500)
Supplement: Supplementary file 1 [file Table1.docx]

Supplementary Appendix

**eMethods. Search Strategy**

**A. PubMed (n=174)**

#1 Colorectal cancer[tiab]

#2 colorectal carcinoma[tiab]

#3 colorectal adenocarcinoma[tiab]

#4 Colon cancer[tiab]

#5 rectal cancer[tiab]

#6 colon adenocarcinoma[tiab]

#7 colon neoplasm[tiab]

#8 rectal adenocarcinoma[tiab]

#9 rectal neoplasm[tiab]

#10 #1 OR #2 OR #3 OR #4 OR #5 OR #6 OR #7 OR #8 OR #9

#11 Immunotherapy[tiab]

#12 PD-1 inhibitor[tiab]

#13 PD-L1 inhibitor[tiab]

#14 CTLA-4 inhibitor[tiab]

#15 Immune checkpoint inhibitor[tiab]

#16 Immune checkpoint blockade[tiab]

#17 Programmed death 1 inhibitor[tiab]

#18 Anti-PD-1[tiab]

#19 Anti-Programmed Cell Death 1[tiab]

#20 Programmed death ligand 1 inhibitor[tiab]

#21 Anti-PD-L1[tiab]

#22 Anti-Programmed Cell Death Ligand-1[tiab]

#23 Checkpoint Inhibitor[tiab]

#24 Checkpoint blockade[tiab]

#25 Programmed Cell Death 1 Receptor[tiab]

#26 Nivolumab[tiab]

#27 Pembrolizumab[tiab]

#28 Dostarlimab[tiab]

#29 Toripalimab[tiab]

#30 Cemiplimab[tiab]

#31 Sintilimab[tiab]

#32 Sulielizumab[tiab]

#33 Envorafumab[tiab]

#34 Atezolizumab[tiab]

#35 Durvalumab[tiab]

#36 Avelumab[tiab]

#37 Ipilimumab[tiab]

#38 #11 OR #12 OR #13 OR #14 OR #15 OR #16 OR #17 OR #18 OR #19 OR #20 OR #21 OR #22 OR #23 OR #24 OR #25 OR #26 OR #27 OR #28 OR #29 OR #30 OR #31 OR #32 OR #33 OR #34 OR #35 OR #36 OR #37

#39 Pathology[tiab]

#40 Pathological[tiab]

#41 Pathologic[tiab]

#42 #39 OR #40 OR #41

#43 neoadjuvant[tiab]

#44 preoperative[tiab]

#45 presurgical[tiab]

#46 presurgery[tiab]

#47 preresection[tiab]

#48 preresectional[tiab]

#49 preoperation[tiab]

#50 preoperations[tiab]

#51 pre-operative[tiab]

#52 pre-surgical[tiab]

#53 pre-surgery[tiab]

#54 pre-operation[tiab]

#55 pre-operations[tiab]

#56 pre-resection[tiab]

#57 before surgery[tiab]

#58 before resection[tiab]

#59 before operation[tiab]

#60 prior to resection[tiab]

#61 prior to surgery[tiab]

#62 prior to operation[tiab]

#63 #43 OR #44 OR #45 OR #46 OR #47 OR #48 OR #49 OR #50 OR #51 OR #52 OR #53 OR #54 OR #55 OR #56 OR #57 OR #58 OR #59 OR #60 OR #61 OR #62

#64 #10 AND #38 AND #42 AND #63

**B. EMBASE (n=254)**

#1 'colorectal cancer':ti,ab,kw

#2 'colorectal carcinoma':ti,ab,kw

#3 'colorectal adenocarcinoma':ti,ab,kw

#4 'colon cancer':ti,ab,kw

#5 'rectal cancer':ti,ab,kw

#6 'colon adenocarcinoma':ti,ab,kw

#7 'colon neoplasm':ti,ab,kw

#8 'rectal adenocarcinoma':ti,ab,kw

#9 'rectal neoplasm':ti,ab,kw

#10 #1 OR #2 OR #3 OR #4 OR #5 OR #6 OR #7 OR #8 OR #9

#11 'immunotherapy':ti,ab,kw

#12 'pd-1 inhibitor':ti,ab,kw

#13 'pd-l1 inhibitor':ti,ab,kw

#14 'ctla-4 inhibitor':ti,ab,kw

#15 'immune checkpoint inhibitor':ti,ab,kw

#16 'immune checkpoint blockade':ti,ab,kw

#17 'programmed death 1 inhibitor':ti,ab,kw

#18 'anti-pd-1':ti,ab,kw

#19 'anti-programmed cell death 1':ti,ab,kw

#20 'programmed death ligand 1 inhibitor':ti,ab,kw

#21 'anti-pd-l1':ti,ab,kw

#22 'anti-programmed cell death ligand-1':ti,ab,kw

#23 'checkpoint inhibitor':ti,ab,kw

#24 'checkpoint blockade':ti,ab,kw

#25 'programmed cell death 1 receptor':ti,ab,kw

#26 'nivolumab':ti,ab,kw

#27 'pembrolizumab':ti,ab,kw

#28 'dostarlimab':ti,ab,kw

#29 'toripalimab':ti,ab,kw

#30 'cemiplimab':ti,ab,kw

#31 'sintilimab':ti,ab,kw

#32 'sulielizumab':ti,ab,kw

#33 'envorafumab':ti,ab,kw

#34 'atezolizumab':ti,ab,kw

#35 'durvalumab':ti,ab,kw

#36 'avelumab':ti,ab,kw

#37 'ipilimumab':ti,ab,kw

#38 #11 OR #12 OR #13 OR #14 OR #15 OR #16 OR #17 OR #18 OR #19 OR #20 OR #21 OR #22 OR #23 OR #24 OR #25 OR #26 OR #27 OR #28 OR #29 OR #30 OR #31 OR #32 OR #33 OR #34 OR #35 OR #36 OR #37

#39 'pathology':ti,ab,kw

#40 'pathological':ti,ab,kw

#41 #39 OR #40

#42 'neoadjuvant':ti,ab,kw

#43 'preoperative':ti,ab,kw

#44 'presurgical':ti,ab,kw

#45 'presurgery':ti,ab,kw

#46 'preresection':ti,ab,kw

#47 'preresectional':ti,ab,kw

#48 'preoperation':ti,ab,kw

#49 'preoperations':ti,ab,kw

#50 'pre-operative':ti,ab,kw

#51 'pre-surgical':ti,ab,kw

#52 'pre-surgery':ti,ab,kw

#53 'pre-operation':ti,ab,kw

#54 'pre-operations':ti,ab,kw

#55 'pre-resection':ti,ab,kw

#56 'before surgery':ti,ab,kw

#57 'before resection':ti,ab,kw

#58 'before operation':ti,ab,kw

#59 'prior to resection':ti,ab,kw

#60 'prior to surgery':ti,ab,kw

#61 'prior to operation':ti,ab,kw

#62 #42 OR #43 OR #44 OR #45 OR #46 OR #47 OR #48 OR #49 OR #50 OR #51 OR #52 OR #53 OR #54 OR #55 OR #56 OR #57 OR #58 OR #59 OR #60 OR #61

#63 #10 AND #38 AND #41 AND #62

**C. Web of Science (n=592)**

#1 Colorectal cancer: Topic

#2 colorectal carcinoma: Topic

#3 colorectal adenocarcinoma: Topic

#4 Colon cancer: Topic

#5 rectal cancer: Topic

#6 colon adenocarcinoma: Topic

#7 colon neoplasm: Topic

#8 rectal adenocarcinoma: Topic

#9 rectal neoplasm: Topic

#10 #1 OR #2 OR #3 OR #4 OR #5 OR #6 OR #7 OR #8 OR #9

#11 Immunotherapy: Topic

#12 PD-1 inhibitor: Topic

#13 PD-L1 inhibitor: Topic

#14 CTLA-4 inhibitor: Topic

#15 Immune checkpoint inhibitor: Topic

#16 Immune checkpoint blockade: Topic

#17 Programmed death 1 inhibitor: Topic

#18 Anti-PD-1: Topic

#19 Anti-Programmed Cell Death 1: Topic

#20 Programmed death ligand 1 inhibitor: Topic

#21 Anti-PD-L1: Topic

#22 Anti-Programmed Cell Death Ligand-1: Topic

#23 Checkpoint Inhibitor: Topic

#24 Checkpoint blockade: Topic

#25 Programmed Cell Death 1 Receptor: Topic

#26 PD-L1 inhibitor: Topic

#27 CTLA-4 inhibitor: Topic

#28 Nivolumab: Topic

#29 Pembrolizumab: Topic

#30 Dostarlimab: Topic

#31 Toripalimab: Topic

#32 Cemiplimab: Topic

#33 Sintilimab: Topic

#34 Sulielizumab: Topic

#35 Envorafumab: Topic

#36 Atezolizumab: Topic

#37 Durvalumab: Topic

#38 Avelumab: Topic

#39 Ipilimumab: Topic

#40 #11 OR #12 OR #13 OR #14 OR #15 OR #16 OR #17 OR #18 OR #19 OR #20 OR #21 OR #22 OR #23 OR #24 OR #25 OR #26 OR #27 OR #28 OR #29 OR #30 OR #31 OR #32 OR #33 OR #34 OR #35 OR #36 OR #37 OR #38 OR #39

#41 Pathology: Topic

#42 Pathological: Topic

#43 #41 OR #42

#44 neoadjuvant: Topic

#45 preoperative: Topic

#46 presurgical: Topic

#47 presurgery: Topic

#48 preresection: Topic

#49 preresectional: Topic

#50 preoperation: Topic

#51 preoperations: Topic

#52 pre-operative: Topic

#53 pre-surgical: Topic

#54 pre-surgery: Topic

#55 pre-operation: Topic

#56 pre-operations: Topic

#57 pre-resection: Topic

#58 before surgery: Topic

#59 before resection: Topic

#60 before operation: Topic

#61 prior to resection: Topic

#62 prior to surgery: Topic

#63 prior to operation: Topic

#64 #44 OR #45 OR #46 OR #47 OR #48 OR #49 OR #50 OR #51 OR #52 OR #53 OR #54 OR #55 OR #56 OR #57 OR #58 OR #59 OR #60 OR #61 OR #62 OR #63

#65 #10 AND #40 AND #43 AND #64

#66 #65 AND (DOCTYPE=(Article) )

**eFigure 1A. Risk of bias graph**


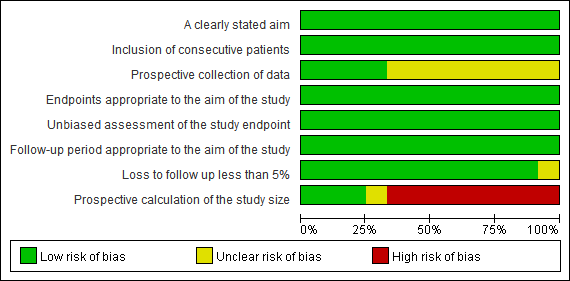


**eFigure 1B. Risk of bias summary**


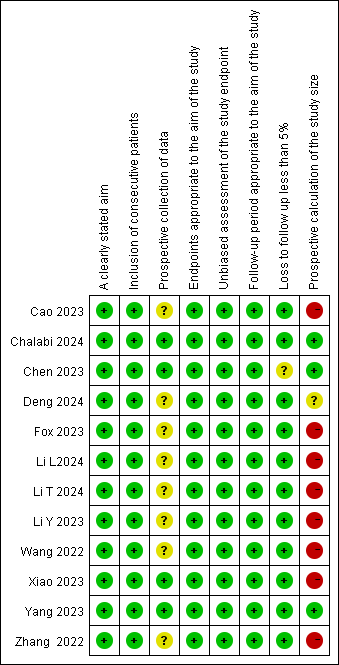


**eFigure 2A. Egger's Test outcome**

**
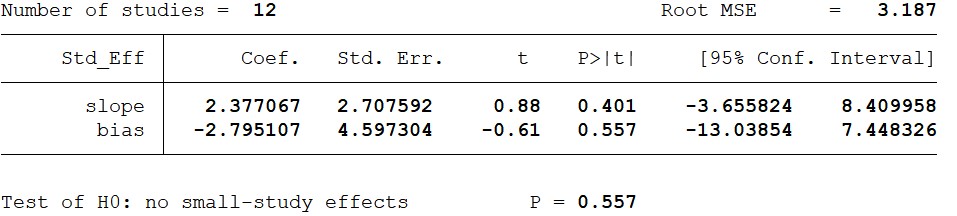
**

**eFigure 2B. Egger's Test plot**

**
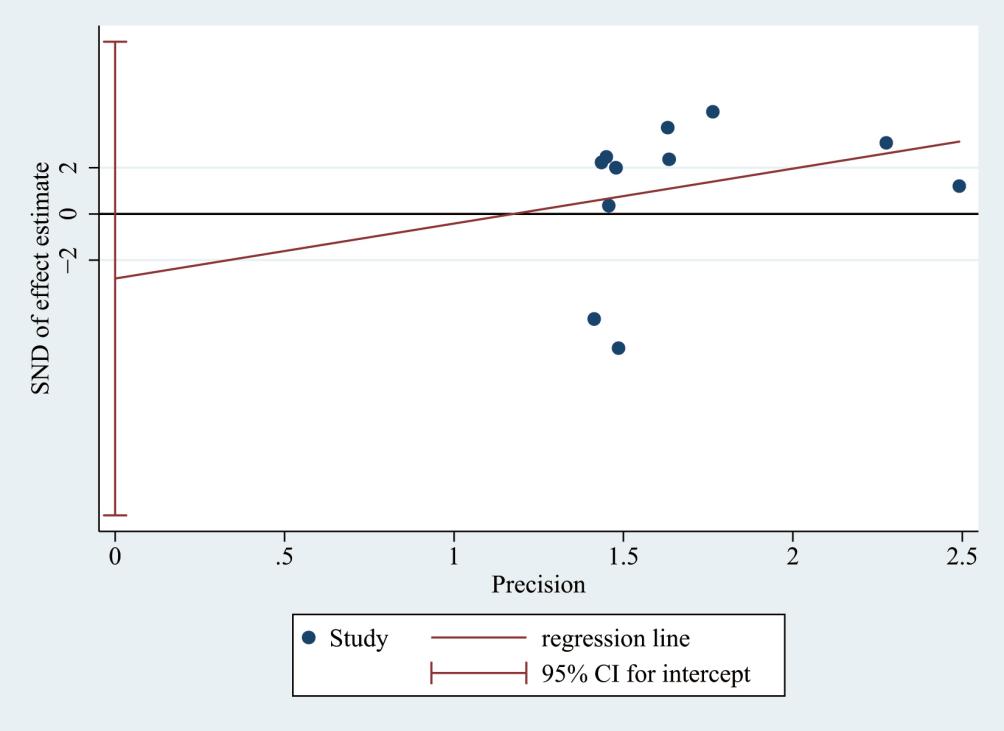
**

**eFigure 2C. Funnel Plot of Total Discordance Between Radiological and Pathological Assessments**


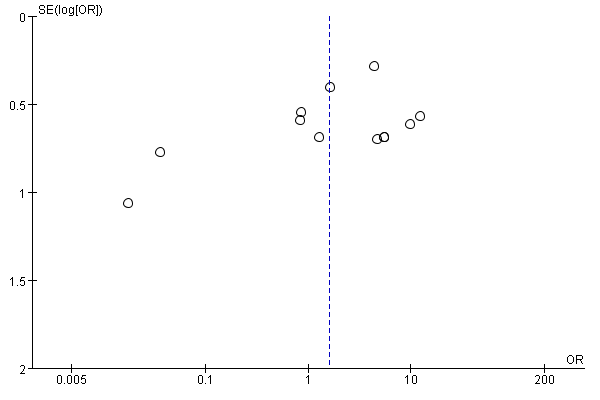


**eFigure 2D. Funnel Plot of Rectal Cancer Discordance**


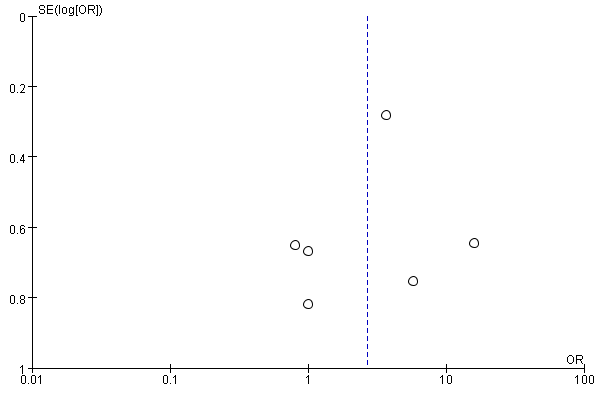


**eFigure 2E. Funnel Plot of Colon Cancer Discordance**


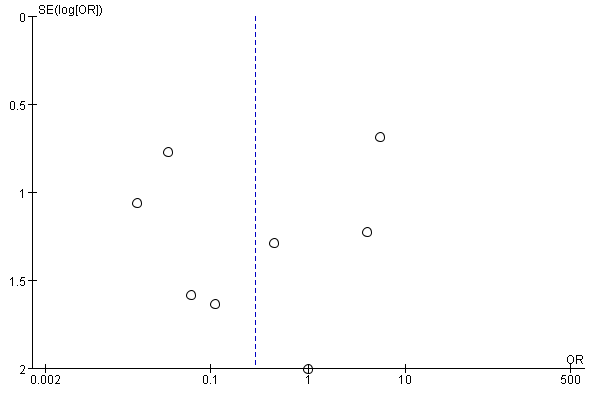


**eFigure 2F. Funnel Plot of Discordance for rectal cancer after re-classification**


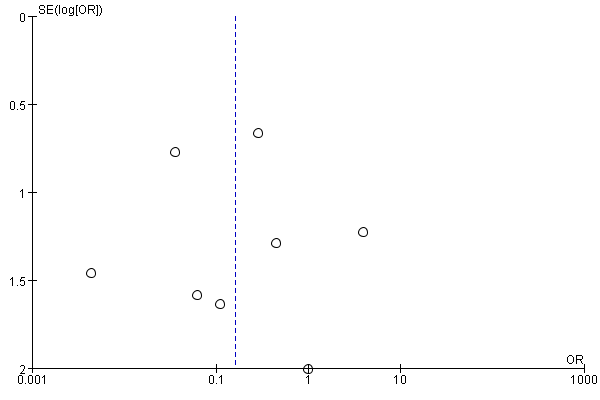


**eFigure 3A. Sensitivity analysis by leave-one-out method**

**
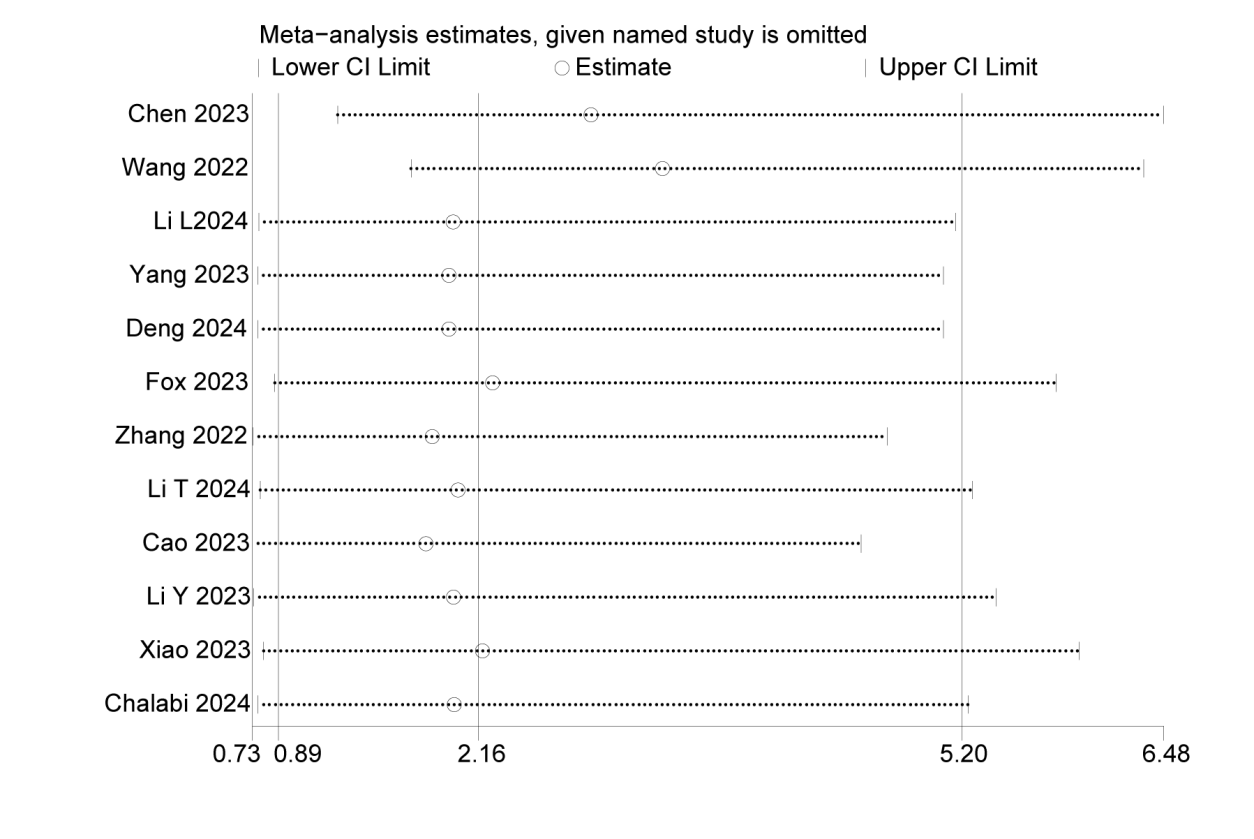
**

**eFigure 3B. Sensitivity analysis after removing Chen et.al and Wang et.al**

**eFigure 4. Pathological Complete Response Among Patients With cCR**


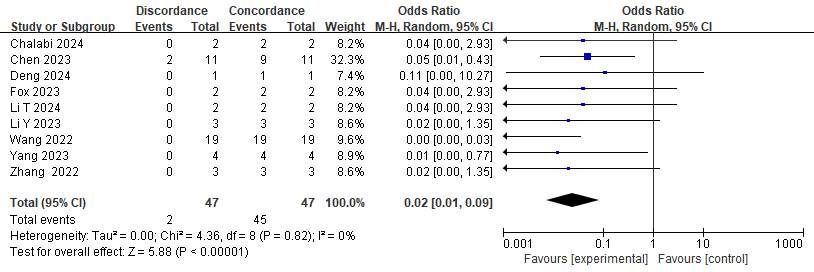


**eFigure 5. Radiological Complete Response Among Patients With pCR**


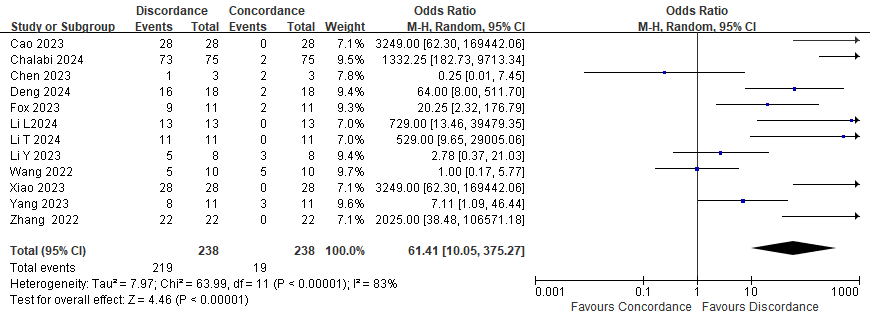


**eTable 1. The details of neoadjuvant immunotherapy plan**

| Study | **Neoadjuvant immunotherapy** |
| --- | --- |
| Xiao et al. [1] | Patients received PD-1 inhibitors (such as pembrolizumab, nivolumab, sintilimab, toripalimab, camrelizumab, and tislelizumab) as neoadjuvant treatment. Most patients (79.5%) received PD-1 inhibitor monotherapy, while others received concurrent therapies, either with ipilimumab (4.1%) or cytotoxic chemotherapy (16.4%, with 2 patients also receiving concurrent radiation). The treatment was administered for at least 2 doses, with radiographic assessment of response generally performed after 2 cycles of treatment and every 2 to 3 months afterward, according to the revised RECIST guideline (version 1.1). |
| Cao et al. [2] | Patients received nivolumab 3 mg/kg was administered intravenously over 30 minutes on day 1 and day 15 of the treatment cycle. Additionally, a single dose of ipilimumab 1 mg/kg was given intravenously on day 1. This regimen was delivered over a 4-week period, with surgery planned within 6 weeks after study enrollment. |
| Chalab et al. [3] | Patients received neoadjuvant immunotherapy with nivolumab (3 mg/kg) and ipilimumab (1 mg/kg). Nivolumab was administered intravenously over 30 minutes on day 1 and day 15 of each 2-week cycle, while ipilimumab was given as a single intravenous dose on day 1. This 4-week treatment regimen was followed by surgical resection planned within 6 weeks after the last neoadjuvant dose. |
| Chen et al. [4] | Patients received neoadjuvant therapy with sintilimab (200 mg intravenously every 21 days). After four initial cycles, patients could choose total mesorectal excision surgery followed by adjuvant sintilimab with or without CapeOX chemotherapy, or continue with another four cycles of sintilimab followed by radical surgery or observation (W＆W strategy for those with clinical complete response). |
| Deng et al. [5] | Patients received either PD-1 blockade monotherapy (200 mg intravenously every 3 weeks) or a combination of nivolumab (3 mg/kg) and ipilimumab (1 mg/kg) according to the NICHE-2 study protocol. Patients receiving monotherapy continued treatment until tumor regression sufficient for radical resection was achieved, while those receiving the combination underwent surgery after two treatment cycles. |
| Fox et al. [6] | Patients received at least one cycle of pembrolizumab (n = 36), nivolumab (n = 1), or vudalimab (anti-PD-1 and anti-CTLA-4 bispecific antibody) (n = 1). The duration of treatment was at the discretion of the treatment team, with a preference for at least 6 months when feasible. |
| Li L et al. [7] | Patients received at least 1 dose of PD-1 blockade (usually 200 mg intravenously over 30 minutes on day 1, every 21 days), with or without CapOx chemotherapy (oxaliplatin 130 mg/m² on day 1 and capecitabine 1000 mg/m² twice daily on days 1 to 14, repeated every 3 weeks). The median number of treatment cycles was 4 (range, 1–8). Efficacy evaluation was performed every 1 or 3 cycles, including CT scan and/or MRI, PET/CT, endoscopy, and measurement of tumor markers (CEA and carbohydrate antigen 19-9). Surgery was planned to be performed within 4 weeks after the last neoadjuvant immunotherapy cycle. For patients who achieved cCR, a watch-and-wait strategy was adopted. |
| Li T et al. [8] | Patients received neoadjuvant single-agent PD-1 blockade immunotherapy. The treatment involved administering PD-1 inhibitors, typically at a dose of 200 mg intravenously over 30 minutes every 21 days. The therapy was continued until the patients achieved a cCR or underwent surgery. |
| Li Y et al. [9] | Patients received PD-1 blockade (200 mg intravenously over 30 minutes on day 1 of each 21-day cycle), with or without CapOx chemotherapy (oxaliplatin 130 mg/m² on day 1 and capecitabine 1000 mg/m² twice daily on days 1–14, repeated every 3 weeks). The primary tumor response was assessed according to the iRECIST criteria, and acute toxicity was graded according to the NCI Common Terminology Criteria for Adverse Events 4.0. After every one or two cycles of neoadjuvant immunotherapy, all patients underwent complete assessment, including CT, magnetic resonance imaging, PET-CT, blood counts, renal biochemistry, hepatobiliary function, thyroid function, cardiac function, and tumor markers (carcinoembryonic antigen and carbohydrate antigen 19-9) to evaluate the general condition and treatment response. The determination of cCR was based on the Memorial Sloan Kettering Cancer Center standard and International Watch & Wait Database criteria. Pathological staging was based on the 8th edition of the American Joint Committee on Cancer TNM staging system. Postoperative complications were classified according to the Clavien–Dindo classification. Surgery was performed 8 to 12 weeks after the last dose of immunotherapy. For patients who achieved cCR, a watch-and-wait strategy was adopted. |
| Wang et al. [10] | Patients received various anti-PD-1 agents, including pembrolizumab, sintilimab, toripalimab, camrelizumab, and nivolumab, either as monotherapy or in combination with other treatments such as ipilimumab, chemotherapy (CapeOx or FOLFOXIRI), or apatinib. The median number of anti-PD-1 antibody cycles before achieving cCR was five (range 1–9). The median time from the start of treatment to cCR was 3.8 months (range 0.7–6.5 months). After achieving cCR, some patients continued to receive anti-PD-1 immunotherapy for a total duration of 6.4 months (range 1.2–26.6 months) until the time of analysis. Tumour response was evaluated according to the RECIST criteria v1.1 after every two to three cycles of anti-PD-1 immunotherapy. Clinical complete response was defined as the absence of residual tumour on digital rectal examination, colonoscopy, pelvic MRI, accompanied by a normal CEA level and negative findings in the chest and abdominal CT scan. |
| Yang et al. [11] | All 20 eligible patients received NIT with a single-agent PD-1 inhibitor. Specifically, 3 patients were treated with pembrolizumab, 9 with sintilimab, and 8 with tislelizumab. On the first day of each treatment cycle, patients received an intravenous infusion of 200 mg of the PD-1 inhibitor. Each treatment cycle lasted for 3 weeks, regardless of the number of cycles administered (200 mg IV Q3W). Prior to initiating nIT, patients did not receive any neoadjuvant chemotherapy (nCT) or chemoradiotherapy (nCRT). Following nIT, 13 of the 20 patients underwent radical resection, while the remaining 7 patients adopted a watch-and-wait strategy. Among the 13 patients who underwent surgery, 9 received adjuvant immunotherapy with PD-1 blockade. |
| Zhang et al. [12] | During the initial phase of treatment, patients received neoadjuvant immunotherapy with a PD-1 inhibitor. Prior to the initiation of immunotherapy, patients underwent comprehensive baseline assessments, including serum carcinoembryonic antigen (CEA) testing, colonoscopy, pathological biopsy, and imaging studies such as chest/abdomen/pelvic enhanced CT and pelvic high-resolution MRI. The primary tumor location, clinical T and N stages, and histological appearance were carefully documented. Treatment volumes were defined as follows: GTV included all visible tumor extent and enlarged lymph nodes; CTV encompassed the GTV with a 0.5 cm margin extension and all regional lymph nodes; PTV was a 1 cm expansion of the CTV in all directions. After neoadjuvant immunotherapy, patients underwent radical surgery. For those who achieved cCR, a watch-and-wait strategy was adopted. Postoperatively, patients received adjuvant immunotherapy with PD-1 blockade. The treatment regimen was meticulously planned and executed to ensure optimal therapeutic outcomes while closely monitoring for any adverse events. |

Notes: dMMR: deficient mismatch repair; MSI-H: microsatellite instability-high; MSI-L: microsatellite instability-low; pMMR: proficient mismatch repair; MSS: microsatellite stable; CRM: circumferential resection margin; EMVI: extramural venous invasion; pCR: pathological complete response; MPR: major pathological response; ORR: objective response rate; cCR: complete clinical response; SBRT: stereotactic body radiation therapy; IMRT: implementation of intensity-modulated radiotherapy; NR: no record.

**eTable 2：Distribution of Neoadjuvant Immunotherapy Regimens**

| **Study** | **PD-1 monotherapy** | **CTL4**  **monotherapy** | **PD-1+CTL4** | **Immune-based Combination Therapy** |
| --- | --- | --- | --- | --- |
| Xiao et al. [1] | 58 | 0 | 3 | 12 |
| Cao et al. [2] | 32 | NR | NR | NR |
| Chalab et al. [3] | 0 | 0 | 115 | 0 |
| Chen et al. [4] | 17 | 0 | 0 | 0 |
| Deng et al. [5] | 8 | 0 | 12 | 5 |
| Fox et al. [6] | 37 | 0 | 1 | 9 |
| Li L et al. [7] | 21 | 0 | 0 | 0 |
| Li T et al. [8] | 23 | 0 | 0 | 0 |
| Li Y et al. [9] | 27 | 0 | 0 | 9 |
| Wang et al. [10] | 11 | 0 | 3 | 5 |
| Yang et al. [11] | 20 | 0 | NR | NR |
| Zhang et al. [12] | 32 | 0 | 0 | 0 |

**eTable 3：Risk of bias in included trials using Methodological Index for Non-randomized Studies（MINORS）**

| **Study** | **D1** | **D2** | **D3** | **D4** | **D5** | **D6** | **D7** | **D8** | **Quality score** |
| --- | --- | --- | --- | --- | --- | --- | --- | --- | --- |
| Xiao et al. [1] | ★★ | ★★ | ★★ | ★★ | ★★ | ★★ | ★★ | NA | 14 |
| Cao et al. [2] | ★★ | ★★ | ★ | ★★ | ★★ | ★★ | ★ | ★★ | 14 |
| Chalab et al. [3] | ★★ | ★★ | ★★ | ★★ | ★★ | ★★ | ★★ | ★★ | 16 |
| Chen et al. [4] | ★★ | ★★ | ★ | ★★ | ★★ | ★★ | ★ | ★★ | 14 |
| Deng et al. [5] | ★★ | ★★ | ★ | ★★ | ★★ | ★★ | ★ | ★ | 13 |
| Fox et al. [6] | ★★ | ★★ | ★ | ★★ | ★★ | ★★ | ★★ | NA | 13 |
| Li Let al. [7] | ★★ | ★★ | ★ | ★★ | ★★ | ★★ | ★★ | NA | 13 |
| Li T et al. [8] | ★★ | ★★ | ★ | ★★ | ★★ | ★★ | ★★ | NA | 13 |
| Li Y et al. [9] | ★★ | ★★ | ★ | ★★ | ★★ | ★★ | ★ | NA | 14 |
| Wang et al. [10] | ★★ | ★★ | ★ | ★★ | ★★ | ★★ | ★★ | NA | 13 |
| Yang et al. [11] | ★★ | ★★ | ★★ | ★★ | ★★ | ★★ | ★ | ★★ | 15 |
| Zhang et al. [12] | ★★ | ★★ | ★ | ★★ | ★★ | ★★ | ★★ | NA | 13 |

**Domains**

D1. A clearly stated aim

D2. Inclusion of consecutive patients

D3. Prospective collection of data

D4. Endpoints appropriate to the aim of the study

D5. Unbiased assessment of the study endpoint

D6. Follow-up period appropriate to the aim of the study

D7. Loss to follow up less than 5%

D8. Prospective calculation of the study size

**References**

1.Xiao BY, Zhang X, Cao TY, Li DD, Jiang W, Kong LH, Tang JH, Han K, Zhang CZ, Mei WJ, Xiao J, Pan ZZ, Li YF, Zhang XS, Ding PR. Neoadjuvant Immunotherapy Leads to Major Response and Low Recurrence in Localized Mismatch Repair-Deficient Colorectal Cancer. J Natl Compr Canc Netw. 2023 Jan;21(1):60-66.e5. doi: 10.6004/jnccn.2022.7060. PMID: 36630898.

2.Cao W, Hu H, Li J, Wu Q, Shi L, Li B, Zhou J, Wang X, Chen J, Wang C, Wang H, Deng W, Huang Y, Deng Y. China special issue on gastrointestinal tumors-Radiological features of pathological complete response in mismatch repair deficient colorectal cancer after neoadjuvant PD-1 blockade: A post hoc analysis of the PICC phase II trial. Int J Cancer. 2023 Dec 1;153(11):1894-1903. doi: 10.1002/ijc.34647. Epub 2023 Jul 6. PMID: 37409565.

3.Chalabi M, Verschoor YL, Tan PB, Balduzzi S, Van Lent AU, Grootscholten C, Dokter S, Büller NV, Grotenhuis BA, Kuhlmann K, Burger JW, Huibregtse IL, Aukema TS, Hendriks ER, Oosterling SJ, Snaebjornsson P, Voest EE, Wessels LF, Beets-Tan RG, Van Leerdam ME, Schumacher TN, van den Berg JG, Beets GL, Haanen JB. Neoadjuvant Immunotherapy in Locally Advanced Mismatch Repair-Deficient Colon Cancer. N Engl J Med. 2024 Jun 6;390(21):1949-1958. doi: 10.1056/NEJMoa2400634. PMID: 38838311.

4.Chen G, Jin Y, Guan WL, Zhang RX, Xiao WW, Cai PQ, Liu M, Lin JZ, Wang FL, Li C, Quan TT, Xi SY, Zhang HZ, Pan ZZ, Wang F, Xu RH. Neoadjuvant PD-1 blockade with sintilimab in mismatch-repair deficient, locally advanced rectal cancer: an open-label, single-centre phase 2 study. Lancet Gastroenterol Hepatol. 2023 May;8(5):422-431. doi: 10.1016/S2468-1253(22)00439-3. Epub 2023 Mar 1. PMID: 36870360.

5.Deng Z, Luo Y, Chen X, Pan T, Rui Y, Hu H, Yan J, Zhang K, Luo C, Song B. Pathological response following neoadjuvant immunotherapy and imaging characteristics in dMMR/MSI-H locally advanced colorectal cancer. Front Immunol. 2024 Sep 27;15:1466497. doi: 10.3389/fimmu.2024.1466497. PMID: 39399495; PMCID: PMC11466765.

6.Fox DA, Bhamidipati D, Konishi T, Kaur H, You N, Raghav KPS, Ge PS, Messick C, Johnson B, Morris VK, Thomas JV, Shah P, Bednarski BK, Kopetz S, Chang GJ, Ludford K, Higbie VS, Overman MJ. Endoscopic and imaging outcomes of PD-1 therapy in localised dMMR colorectal cancer. Eur J Cancer. 2023 Nov;194:113356. doi: 10.1016/j.ejca.2023.113356. Epub 2023 Sep 22. PMID: 37827065.

7.Li Y, Tan L, Chen N, Liu X, Liang F, Yao Y, Zhang X, Wu A. Neoadjuvant Immunotherapy Alone for Patients With Locally Advanced and Resectable Metastatic Colorectal Cancer of dMMR/MSI-H Status. Dis Colon Rectum. 2024 Nov 1;67(11):1413-1422. doi: 10.1097/DCR.0000000000003290. Epub 2024 Sep 11. PMID: 39260435.

8.Li YJ, Liu XZ, Yao YF, Chen N, Li ZW, Zhang XY, Lin XF, Wu AW. Efficacy and safety of preoperative immunotherapy in patients with mismatch repair-deficient or microsatellite instability-high gastrointestinal malignancies. World J Gastrointest Surg. 2023 Feb 27;15(2):222-233. doi: 10.4240/wjgs.v15.i2.222. PMID: 36896306; PMCID: PMC9988634.

9.Li Y, Liang F, Li Z, Zhang X, Wu A. Neoadjuvant Immunotherapy for Patients With Microsatellite Instability-High or POLE-Mutated Locally Advanced Colorectal Cancer With Bulky Tumors: New Optimization Strategy. Clin Colorectal Cancer. 2025 Mar;24(1):18-31.e2. doi: 10.1016/j.clcc.2024.07.001. Epub 2024 Jul 9. PMID: 39095269.

Wang QX, Xiao BY, Cheng Y, Wu AW, Zhang T, Wang H, Zhang X, Huang WX, Tang JH, Jiang W, Steele SR, Krishnamurthi S, Li Y, Cai J, Kong LH, Li DD, Pan ZZ, Zhang XS, Ding PR. Anti-PD-1-based immunotherapy as curative-intent treatment in dMMR/MSI-H rectal cancer: A multicentre cohort study. Eur J Cancer. 2022 Oct;174:176-184. doi: 10.1016/j.ejca.2022.07.016. Epub 2022 Aug 26. PMID: 36030556.

11.Yang R, Wu T, Yu J, Cai X, Li G, Li X, Huang W, Zhang Y, Wang Y, Yang X, Ren Y, Hu R, Feng Q, Ding P, Zhang X, Li Y. Locally advanced rectal cancer with dMMR/MSI-H may be excused from surgery after neoadjuvant anti-PD-1 monotherapy: a multiple-center, cohort study. Front Immunol. 2023 Jun 27;14:1182299. doi: 10.3389/fimmu.2023.1182299. PMID: 37441082; PMCID: PMC10333582.

12.Zhang X, Yang R, Wu T, Cai X, Li G, Yu K, Li Y, Ding R, Dong C, Li J, Hu R, Feng Q, Li Y. Efficacy and Safety of Neoadjuvant Monoimmunotherapy With PD-1 Inhibitor for dMMR/MSI⁃H Locally Advanced Colorectal Cancer: A Single-Center Real-World Study. Front Immunol. 2022 Jul 25;13:913483. doi: 10.3389/fimmu.2022.913483. PMID: 35958603; PMCID: PMC9359076.
